# Genome Size Evolution Differs Between *Drosophila* Subgenera with Striking Differences in Male and Female Genome Size in *Sophophora*

**DOI:** 10.1534/g3.119.400560

**Published:** 2019-07-29

**Authors:** Carl E. Hjelmen, Heath Blackmon, V. Renee Holmes, Crystal G. Burrus, J. Spencer Johnston

**Affiliations:** * Department of Biology and; † Department of Entomology, Texas A&M University, College Station, TX 77843

**Keywords:** Genome size, sex chromosome, *Drosophila*, phylogenetic comparative methods

## Abstract

Genome size varies across the tree of life, with no clear correlation to organismal complexity or coding sequence, but with differences in non-coding regions. Phylogenetic methods have recently been incorporated to further disentangle this enigma, yet most of these studies have focused on widely diverged species. Few have compared patterns of genome size change in closely related species with known structural differences in the genome. As a consequence, the relationship between genome size and differences in chromosome number or inter-sexual differences attributed to XY systems are largely unstudied. We hypothesize that structural differences associated with chromosome number and X-Y chromosome differentiation, should result in differing rates and patterns of genome size change. In this study, we utilize the subgenera within the *Drosophila* to ask if patterns and rates of genome size change differ between closely related species with differences in chromosome numbers and states of the XY system. Genome sizes for males and females of 152 species are used to answer these questions (with 92 newly added or updated estimates). While we find no relationship between chromosome number and genome size or chromosome number and inter-sexual differences in genome size, we find evidence for differing patterns of genome size change between the subgenera, and increasing rates of change throughout time. Estimated shifts in rates of change in sex differences in genome size occur more often in *Sophophora* and correspond to known neo-sex events.

Genome size varies widely across the tree of life, with no clear correlation to organismal complexity (Mirskey and Ris 1951; Gregory 2001; Palazzo and Gregory 2014). This extreme variation is therefore not attributed to coding sequences in eukaryotes, but rather to differences in non-coding regions such as introns, and inflation of the genome via TEs and repetitive DNA (Gregory and Hebert 1999; Kidwell 2002; Kelley *et al.* 2014; Sessegolo *et al.* 2016; Wright 2017). Among closely related species of plants and closely related *Drosophila* species, much of the variation in genome size has been explained by the differential accumulation of transposable elements (Bennetzen and Kellogg 1997; Ågren and Wright 2011; Śliwińska *et al.* 2016). For example, *Drosophila melanogaster* has a significantly greater accumulation of transposable elements in comparison to *D. simulans*, and has a larger genome size (Vieira and Biemont 2004). The same pattern of increased transposable element content with increased genome size was found in the larger *Drosophila melanogaster* species subgroup (Boulesteix *et al.* 2005). This pattern of increased transposable element load with increased genome size, has been shown to be significant, when analyzed in a phylogenetic manner across 26 species of the *Drosophila* genus (Sessegolo *et al.* 2016).

Recent work that looks at genome size evolution in a phylogenetic context, finds various patterns for change (Arnqvist *et al.* 2015; Jeffery *et al.* 2016; Sessegolo *et al.* 2016; Hjelmen and Johnston 2017; Lower *et al.* 2017). Most of these have focused on widely-diverged species. Little work has been done to compare the patterns of genome size change in closely related species with known structural differences in genomes. In particular, variation in genome size due to differences in chromosome number among related species, and inter-sexual differences due to differentiation of the largely heterochromatic Y chromosome in XY systems are largely unstudied.

While increasing chromosome number may hypothetically increase repetitive, heterochromatic regions such as centromeres and telomeres (Levis *et al.* 1993; Sun *et al.* 1997) and therefore genome size, to date, little to no evidence of relationship between genome size and chromosome number exists (Jeffery *et al.* 2012; Elliott and Gregory 2015; Slijepcevic 2018). However, the structural differences associated with varying chromosome number, and the resulting addition of repetitive regions, may result in differing rates and patterns for genome size change. Hypothetically, genomes with more baseline repetitive regions may be more likely to see expansions or contractions in the genome. In this vein, the structural differences in heteromorphic sex chromosome systems allow for differing patterns of change between sexes due to loss of genic material and physical DNA or bloating of the chromosome through increased mobile element activity (Zhou *et al.* 2012). In this study, we utilize the subgenera within the *Drosophila* to ask if patterns and rates of genome size change differ between closely related species with differences in chromosome number and states of evolution of the XY system including known origins of neo sex chromosome events.

Species in the genus *Drosophila* have been the subject of a wide variety of biological studies, including phylogenetics and genome size (Gregory and Johnston 2008; Van Der Linde and Houle 2008; Van Der Linde *et al.* 2010; Hjelmen and Johnston 2017; Hjelmen *et al.* 2019). The wealth of information available for this genus allows researchers to develop very ambitious large scale evolutionary studies with relative ease. Importantly this genus is separated into subgenera, *Sophophora* and *Drosophila*, which diverged an estimated 40-65 million years ago (Russo *et al.* 1995; Tamura *et al.* 2004; Obbard *et al.* 2012). The two subgenera can largely be separated karyotypically. The majority of *Drosophila* subgenus species have the proposed ancestral 6 telocentric chromosome karyotype whereas many *Sophophora* subgenus species have a reduced chromosome number due to fusion events that formed large metacentric autosomes (Reviewed in Schulze *et al.* 2006). A comparison between the subgenera provides both biological replication and a test for the effect of the change in chromosome number.

Although the *Drosophila* genus has been widely studied, much of the emphasis for genome size studies has been placed on species within *Sophophora* (120 records on genomesize.com, for ∼300 species in the subgenus), the subgenus which includes the very well-studied *D. melanogaster* (Boulesteix *et al.* 2005; Gregory and Johnston 2008; Hjelmen and Johnston 2017; Hjelmen *et al.* 2019). In comparison, the subgenus *Drosophila* has been dramatically underrepresented (52 records on genomesize.com, for the ∼1400 species in the subgenus) with few studies and low numbers of representative taxa (Schulze and Lee 1986; Bosco *et al.* 2007; Gregory and Johnston 2008). Therefore we update or estimate anew the genome size for females and males of 92 species (53 new species genome size records, 39 updated species genome sizes), with a focus on the *Drosophila* subgenus, including *Zaprionus*. In order to analyze our data in a comparative framework we infer a phylogeny allowing confident placement of each species within each subgenus. With an understanding of the phylogenetic relationships, we make comparisons of genome size variation between the subgenera and analyze the evolutionary dynamics of genome size evolution between species in the subgenera and across the genus as a whole. *Sophophora* genome size has been shown to best fit the accordion model hypothesis of evolution (Hjelmen and Johnston 2017; Hjelmen *et al.* 2019). In the accordion model hypothesis, genome size is allowed to increase, decrease, or maintain genome size in equilibrium in each species with increases in size due to transposable element insertion balanced by decreases due to large segmental deletions (Kapusta *et al.* 2017). These larger insertions and deletions allow increases and decreases in genome size similar to the mutational equilibrium hypothesis (Petrov 2002) at a rate which can account for the differences between species (Gregory 2004). In the *Sophophora*, the accordion model hypothesis is supported with strong phylogenetic signal and mostly gradual change throughout branches the phylogeny.

We examine here the variation of genome size in the subgenus *Drosophila* for comparison to *Sophophora* and the genus as a whole. We hypothesize that, despite 40-65 million years of evolution since the divergence of the subspecies, under the accordion model, there should not be remarkably different patterns between the subgenera. That hypothesis would be rejected if there is evidence for decreased phylogenetic signal in genome size change in the *Drosophila* subgenus or a different rate of change between the subgenera. A difference, if found, could be attributed to the karyotypic difference between these species. A difference could also be attributed to sex chromosome evolution. We investigate the difference in genome size between females and males of each species as an intersexual difference to determine if possible differences in the patterns of sex differentiation in genome size exist between the subgenera. Given the presence of a common XY system in most of these species, we hypothesize that the patterns will be generally the same among subgenera, and be similar to those found in earlier studies (Hjelmen *et al.* 2019). If differing patterns are found, it could suggest differing levels of sex chromosome turnover and differentiation between the subgenera.

## Materials and Methods

### Phylogeny reconstruction

Sequences for 16 genes were downloaded from NCBI GenBank in order to create a molecular phylogeny (4 mitochondrial and 12 nuclear including both structural and protein coding genes) (*COI*, *COII*, *COIII*, *Cytb*, *Amy*, *AmyRel*, *Ddc*, *boss*, *SNF*, *Marf*, *Sod*, *per*, *Wee*, *HB*, *ADH*, *and **fkh*) (accession numbers in Table S1). These sequences were downloaded for 152 species within Drosophilidae, five of which we designated as outgroup species (*Chymomyza amoena*, *C. procnemis*, *Scaptodrosophila stonei*, *S. lebanonensis*, and *S. pattersoni*). These sequences were aligned using MAFFT v.7 online with iterative refinement methods (http://mafft.cbrc.jp/). Amino acid translations of these alignments were inspected in Mesquite for irregularities and corrected by hand as needed.

Each sequence alignment was then analyzed in JModelTest 2.1.4 to determine the model of sequence evolution that produced the best likelihood value (Darriba *et al.* 2012). This likelihood search assumed 11 possible substitution schemes, allowing for invariant sites and gamma distributions. A fixed BIONJ-JC tree was used for all calculations. All genes were found to have the same suggested model for phylogeny reconstruction, a GTR substitution model with gamma distribution and invariant sites.

All sequences were interleaved and concatenated to produce a 10,382 bp alignment. Missing sequence data were input for taxa that did not have gene sequence data for every gene, as per the supermatrix method (Van Der Linde *et al.* 2010). The resulting alignment consisted of 58% missing data. Overall, there was an average of seven genes per taxa, with a minimum of three genes.

A phylogeny for the 152 species was reconstructed utilizing MrBayes 3.2.3 on the CIPRES supercomputer (http://www.phylo.org/) with four chains and four runs and a GTR gamma + I evolutionary model for 44,119,000 generations (sampling every 1,000 generations) using a branch length Dirichlet prior of (1, 0.5, 1, 1,) (Huelsenbeck and Ronquist 2001; Ronquist and Huelsenbeck 2003). Outputs for parameters were visualized in Tracer v 1.6 to assure that runs reached convergence and to determine the appropriate burn-in time. A consensus tree was constructed based on 158,828 trees from the prior distribution was made ultrametric using the penalized likelihood approach in APE (Sanderson 2002; Paradis *et al.* 2004). The lambda smoothing parameter was 1 and was chosen after cross-validation through the chronopl function in the package APE (Paradis *et al.* 2004). This ultrametric tree was scaled so that the split between *Chymomyza* and *Scaptodrosophila* was 70 million years, consistent with the most recent divergence time estimates (Russo *et al.* 2013). In order to analyze the difference between *Sophophora* and *Drosophila*, the phylogeny was trimmed to two smaller trees using the drop.tip function in the package APE (Paradis *et al.* 2004; Team 2016). This produced phylogenies for each of our two subclades of interest. Genome sizes for females and males, as well as the sex differences, were mapped onto the phylogenies using the ContMap function from the phytools package (Revell 2012).

### Genome size estimates

Prior estimates of genome size for 60 *Drosophila* species were found in the published literature (Gregory and Johnston 2008; Hjelmen and Johnston 2017; Hjelmen *et al.* 2019). New genome sizes estimates were produced for 92 additional species of *Drosophila*, *Chymomyza*, *Zaprionus*, *Scaptomyza*, and *Hirtodrosophila*, with a focus in the *Drosophila* subgenus. Individuals for these species were obtained from the UC San Diego Species Stock Center (http://stockcenter.ucsd.edu) and the National *Drosophila* Species Stock Center (http://blogs.cornell.edu/drosophila/) (Table S2). Genome sizes were estimated utilizing flow cytometry (Johnston *et al.* 2019) . Briefly, neural tissue was dissected from samples and placed into 1 mL of Galbraith buffer. All samples were co-prepared with an appropriate standard (yw *D. melanogaster* female = 175 Mbp, Lab strain *D. virilis* female = 328 Mbp). Samples and standards were gently ground with a “loose” Kontes “A” pestle 15 times in order to release nuclei. Samples were then passed through a 41 micrometer filter before staining with 25µl of 1mg/µl propidium iodide. Samples were allowed to incubate for at least 20 min to ensure proper stain saturation had occurred. Samples were then run on a Partec CyFlow SL_3 cytometer with a 532 nm green laser or a Beckman Coulter CytoFlex flow cytometer with a 488 nm blue laser. Samples were run on both flow cytometers initially to ensure no machine differences in results. Means of fluorescence peaks produced by 2C nuclei of both the sample and the standard were determined using statistical gates supplied with the software of each cytometer. Sample preparation was repeated for at least 5 individuals of each sex and species, in order to generate replicates, an average genome size estimate and the standard error of that estimate.

The difference in genome sizes between females and males in each species, or intersexual difference, was calculated by subtracting the 2C male genome size estimate from the 2C female genome size estimate (Hjelmen *et al.* 2019). Positive sex difference values indicate females of the species have a larger genome than that of the male.

### Chromosome count information

Chromosome count information was gathered for species with information from the Tree of Sex Database (Tree Of Sex Consortium 2014). The haploid chromosome count was used to make direct comparisons with the 1C genome size of species.

### Statistical tests

**Figure 1:**
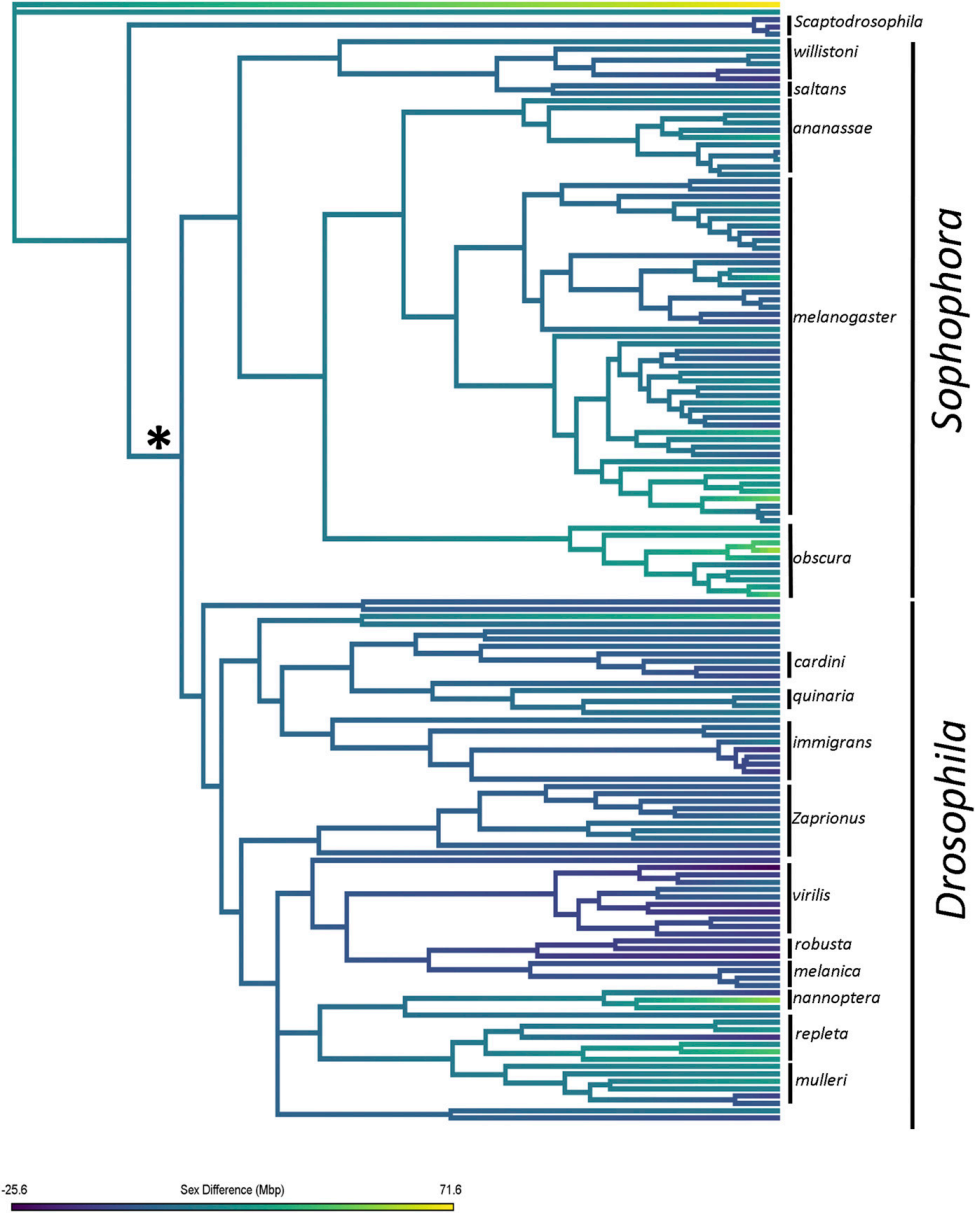
Phylogeny of *Drosophila* genus. The phylogeny of *Drosophila* was reconstructed using a supermatrix method in MrBayes. Posterior support values for each node can be found in Figure S1. Further analyses referring to *Sophophora* and *Drosophila* are based on the taxa's placement in the clades that split at the node indicated with an asterisk. The phylogeny was dated using known divergence times. Here, sex difference for all lineages is reconstructed using a simple Brownian motion model. Negative trait values correspond to species with larger genomes in males whereas positive values correspond to species with larger genomes in females.

In order to test for significant differences between the subgenera, species were placed within the subgenus *Sophophora* or *Drosophila* based on the large split (Figure 1) of the phylogeny into 2 major clades. For example, that *Zaprionus* species were included in the *Drosophila* subgenus data. The *Sophophora* data included 76 species and the *Drosophila* subset included 71 species. Since species outside of the subgenera were not included here, genera such as *Chymomyza* and *Scaptodrosophila* were excluded from comparisons of the subgenera. The variation in genome size was also visualized in histogram format using R 3.3.0 (Team 2016). Using Proc GLM in SAS (Raleigh, NC), a pdiff test was run to test for significant differences between the sexes in each species followed with a Benjamini-Hochberg correction using a conservative false discovery rate of 0.05 (Benjamini and Hochberg 1995). T-tests were used to test for significant differences between the sexes as well as the differences between the subgenera. These tests were run for both female and male genome size, as well as for sex difference. All *t*-tests and histograms were run in R 3.3.0 (Team 2016).

Regression analyses were run to compare each male and female genome size and sex difference to chromosome count for each species. In order to account for phylogeny, a phylogenetic ANOVA was run on these comparisons. P-values for the ANOVAs were calculated based on 1000 simulations. All analyses were completed using the phylANOVA function in the Phytools package in R 3.3.0 (Revell 2012; Team 2016).

### Evolutionary model analyses

The whole genome size of females and males, the intersexual difference in genome size, and the chromosome count were analyzed on the reconstructed phylogeny using the fitContinuous function in the package “geiger” in R 3.3.0 (Harmon *et al.* 2009; Team 2016). This process allows comparison of three different models of trait evolution (Brownian Motion [BM], Ornstein-Uhlenbeck [OU], and white-noise) with likelihood and Akaike Information Criterion (AICc) values. In addition to these models, Pagel’s parameters of evolution were estimated for each variable of interest, female whole genome size, male whole genome size, the intersexual difference in genome size of each species, and chromosome count (Pagel 1999). Each of these analyses were performed with the entire phylogeny including closely related outgroup species, as well as with the reduced *Sophophora* and *Drosophila* phylogenies without the outgroup species. The above analyses allow for the comparison of the patterns, mode, and rate of evolution across the entire genus, and between the subgenera.

Pagel’s parameters of evolution are comprised of three parameters: λ, κ, and δ. The parameter λ tests for phylogenetic signal of the trait of interest across the phylogeny, assuming Brownian motion (λ = 1, strong phylogenetic signal, λ < 1, weak signal). Strong phylogenetic signal indicates that the variation in the trait is explained largely by the evolutionary relationships between the species. The κ parameter tests for a gradual *vs.* punctuational mode of trait evolution (κ = 1, gradual change, κ = 0, punctuated change, κ > 1). Finally, δ tests how the trait evolves along the long paths, or where in the entire phylogeny the change occurs (δ = 1, gradual change, δ < 1, early change in phylogeny/evolutionary rates slowing, δ > 1, late change in tree/increasing rates of change). Each of these analyses were completed utilizing the pgls function in the caper package of R 3.3.0 (Orme 2013). These values were then used in conjunction with the colorized trait-map phylogenies for interpretation of evolutionary patterns.

To complement our estimates of Pagel’s parameters and model testing, we also conducted an analysis using Bayesian Analysis of Macroevolutionary Mixtures (BAMM) where our traits (female whole genome size, male whole genome size, and intersexual difference) evolved by Brownian motion allowing for the possibility of rate shifts in our tree. The priors for the number of shifts were determined for each trait of interest before BAMM analyses using the BAMMtools package in R 3.3.0 (Rabosky *et al.* 2014). Each BAMM analysis was run for 10,000,000 generations with a 10% burn-in to ensure sufficient effective sizes. The coda package in R was used in order to ensure all runs reached convergence (Plummer *et al.* 2006). Credible rate shifts sets were calculated for each trait in order to estimate the most likely number of rate shifts on the phylogeny and to identify the clades in which rate shifts are likely to occur.

In instances of clades with high probability of rates shifts identified by BAMM, the brownie.lite function in phytools was used to perform a censored rate test for significantly different rates of evolution within these clades in comparison to the rest of the phylogeny (O'Meara *et al.* 2006; Revell 2012).

### Data Availability

Accession numbers used for phylogeny reconstruction are available in Table S1. Genome size estimates and sex difference values are available in Table S2. Phylogeny Nexus file are available upon request. Supplemental material available at FigShare: https://doi.org/10.25387/g3.8170847.

## Results

### Phylogeny reconstruction

The overall phylogeny for *Drosophila* is well supported, with the majority of nodes having posterior probabilities of 1.0, with the lowest being 0.56 (Fig. S1). The relationships found in this phylogeny are in agreement with those found in other large phylogenetic studies (Drosophila 12 Genomes Consortium 2007; Da Lage *et al.* 2007; Gregory and Johnston 2008; Van Der Linde *et al.* 2010). Tree scaling by the *Chymomyza/Scaptodrosophila* split has provided species divergences that are supported by literature. For example, our scaling resulted in an estimated ∼4 my divergence of *D. simulans* and *D. sechellia* and ∼2 my divergence between *D. triauraria* and *D. auraria*, and are similar to the estimated 3.3 mya and 2.6 mya splits estimated by Russo *et al.* (2013). Our estimated 12 mya divergence of *D. acanthoptera* and *D. pachea* is very close to the estimated divergence of 13.4 mya from Morales-Hojas and Vieira (2012). This congruence suggests that phylogenetic relationships and branch lengths should be reliable in this reconstructed phylogeny.

### Genome size estimates

Whole genome size information for females, males, and the corresponding intersexual difference are given in Table S2. Overall, *Drosophila* (*Drosophila* and *Sophophora* subgenera, *Chymomyza*, *Hirtodrosophila*, *Samoaia*, *Scaptomyza*, *Scaptodrosophila*, and *Zaprionus*) were found to have a female whole genome size average of 220.6 Mbp and a male whole genome size average of 215.5 Mbp (n = 152). These overall genome sizes ranged by more than 240 Mbp, from 134.7 Mbp (*D. busckii*) to 395.2 Mbp (*C. amoena*) in females and 136.5 Mbp to 384.8 Mbp in males (*D. busckii* and *C. amoena*, respectively). The intersexual difference of each species, which we assume here to be due to the difference in the size of X and Y chromosomes, averaged 9.9 Mbp, indicating that female genomes are larger on average than male genomes (Table 1). In terms of raw estimates, 37 species had males with larger genomes than females, and 112 species had females with larger genomes than males (Table S2).

**Table 1:** Average female and male genome size (Mbp) and intersexual difference by genus

Genus	N	Female Mbp	Male Mbp	Sex Difference
*Chymomyza*	2	346.7	323.6	46.2
*Drosophila*	132	219.5	214.4	9.9
*Drosophila*	57	215.4	212.7	4.7
*Sophophora*	75	222.6	215.7	13.8
*Hirtodrosophila*	2	207.2	193.3	27.9
*Scaptodrosophila*	4	229.4	230.1	−1.3
*Scaptomyza*	1	200.2	193.1	14.2
*Zaprionus*	10	206.5	204.7	3.6
*Samoaia*	1	261.8	260.4	2.8
Grand Total	152	220.6	215.5	9.9

Summary statistics were calculated for the entire data set and for the subspecies *Sophophora* and *Drosophila*. Species were determined to fit within the subgenus *Sophophora* or *Drosophila* based on the large split (Indicated by ‘*’ in Figure 1) of the phylogeny into 2 major clades. This means, for example, *Zaprionus* species are included in the *Drosophila* subgenus. *Sophophora* data included 76 species and *Drosophila* included 71 species.

**Figure 2:**
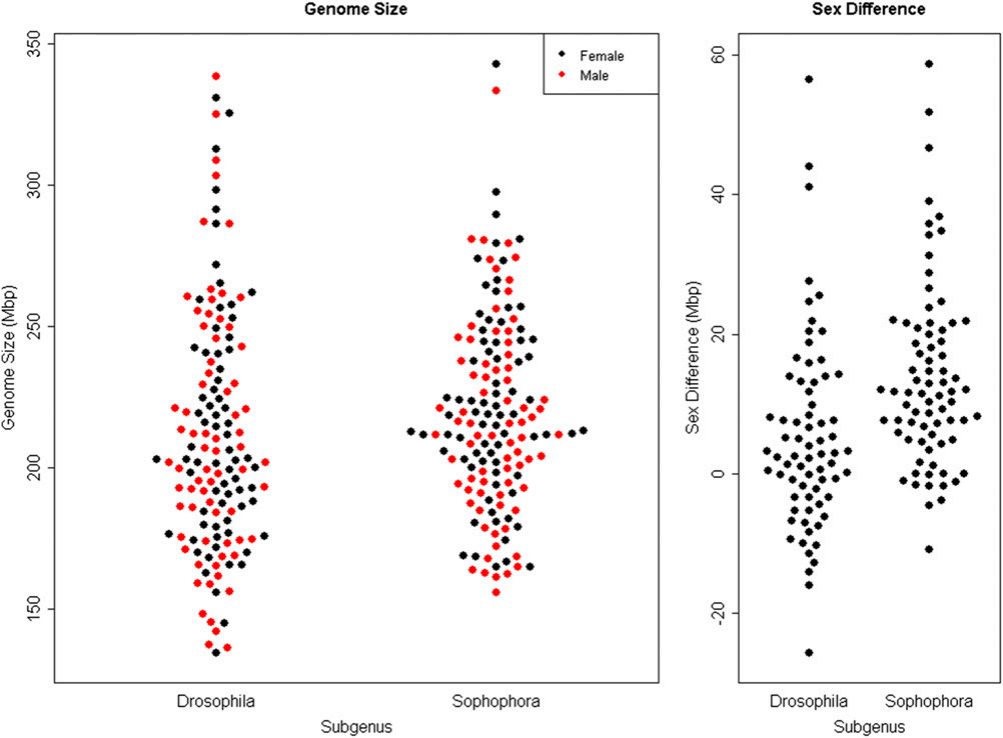
Genome Size by Sex and Sex Difference Calculation between *Sophophora* and *Drosophila* (Left panel) Whole genome size comparisons between *Drosophila* (left) and *Sophophora* (right). Male whole genome sizes are indicated in red. Female whole genome sizes are indicated in black. (Right panel) Intersexual difference comparison between the subgenera.

The whole genome size of the *Sophophora* (including: *Hirtodrosophila duncani*) females and males average 223.0 Mbp and 216.1 Mbp, respectively. *Drosophila* (including: *Scaptomyza*, *Zaprionus*, *Samaoia*, *Scaptodrosophila latifasciaeformis*, and *Hirtodrosophila pictiventris*) females and males average 213.7 Mbp and 210.8 Mbp, respectively (Figure 2). *Sophophora* had an average intersexual difference of 13.9 Mbp while *Drosophila* had an average intersexual difference of 5.1 Mbp. The positive values for the difference indicate that female genomes, on average, are larger than male.

While genome size is not significantly different between the subgenera, the intersexual difference due to the X-Y was found to significantly differ between *Sophophora* and *Drosophila* (*t*-test, t = 3.93, df = 143.24, *P* = 0.0001, Figure 2). There were no significant differences found between the whole genome sizes of the sexes between the subgenera (ANOVA, F = 1.324 on 3 and 290 Df, *P* = 0.2667, Figure 2).

**Figure 3:**
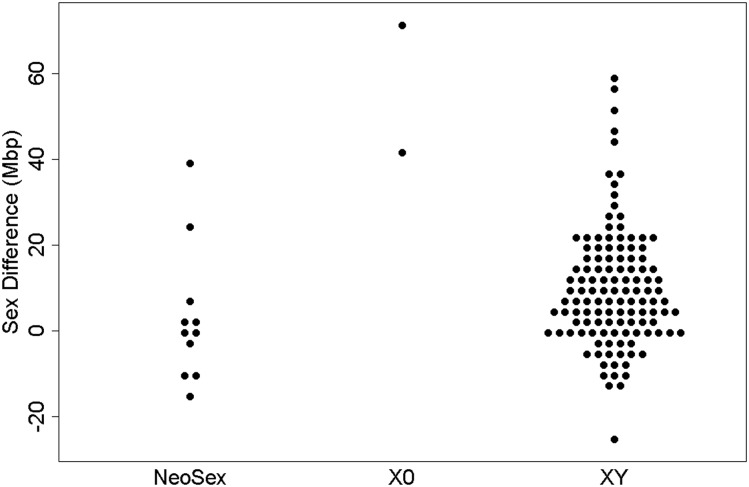
Intersexual difference between documented Neo-Sex, XO, and XY species.

Intersexual differences for three different sex chromosome systems (Neo-Sex, XO, and XY) were compared using generalized linear methods (GLM) in R 3.3.0. Neo-Sex and XO systems were determined by literature review and the tree of sex database (Tree Of Sex Consortium 2014). Information for the strains used for genome size analysis can be found in Table S2. No statistically significant difference was found between Neo-Sex and XY systems (*P* = 0.140, Figure 3). However, the intersexual difference in XO systems is statistically larger than Neo-Sex and XY systems (*P* < 0.0001, Figure 3).

After pdiff analysis with a Benjamini-Hochberg correction for multiple tests, 83 of the 152 analyzed species were found to have statistically significant differences between female and male genome size (Table 2). 45 of these species were grouped within *Sophophora*, 35 were grouped within *Drosophila*, and three were in outgroup species. 11 species were found to have males with statistically significantly larger genomes than the females. 9 of these species were found in the *Drosophila* subgenus, most notably *D. albomicans*, *D. robusta*, and *D. lacertosa*, all of which have reports of neo-sex chromosome systems (Flores *et al.* 2008).

**Table 2:** Species with significantly different Genome Sizes between Sexes. 83 species were found to have statistically different genome sizes between the sexes. In 11 of these species male genome sizes were found to be statistically larger than female genomes (bold)

*Sophophora*			*Drosophila*		
Species	Sex Diff	Significance	Species	Sex Diff	Significance
*D. affinis*	46.6	*P = 0.0001*	*D. acanthoptera*	20.4	*P* = 0.0001
*D. algonquin*	22.0	*P = 0.0001*	***D. albomicans***	**−9.4**	*P* = 0.01538
*D. ambigua*	23.8	*P = 0.0001*	***D. anceps***	**−11.4**	*P* = 0.0065
*D. ananassae*	13.0	*P = 0.0005*	***D. arawakana***	**−5.3**	*P* = 0.0055
*D. auraria*	12.0	*P = 0.0021*	*D. bromeliae*	7.7	*P* = 0.0001
*D. azteca*	20.0	*P = 0.0001*	*D. buzzatii*	21.8	*P = 0.0001*
*D. baimaii*	11.2	*P = 0.0025*	*D. eohydei*	24.6	*P = 0.0001*
*D. barbarae*	20.8	*P = 0.0001*	***D. ezoana***	**−10.0**	*P = 0.0109*
*D. biauraria*	51.8	*P = 0.0001*	*D. guarani*	5.2	*P = 0.0083*
*D. bicornuta*	36.8	*P = 0.0001*	*D. guttifera*	13.1	*P = 0.0001*
*D. bifasciata*	28.8	*P = 0.0001*	*D. hydei*	15.8	*P = 0.0001*
*D. bunnanda*	11.6	*P = 0.0031*	*D. hypocausta*	4.7	*P* = 0.0168
*D. capricorni*	12.9	*P = 0.0009*	***D. kepulauana***	**−12.7**	*P* = 0.0001
*D. emarginata*	13.4	*P = 0.0001*	*D. kohkoa*	16.3	*P = 0.0001*
*D. ficusphila*	18.0	*P = 0.0001*	***D. lacertosa***	**−10.2**	*P = 0.0064*
*D. greeni*	14.8	*P = 0.0001*	*D. limensis*	20.4	*P* = 0.0001
*D. jambulina*	17.2	*P = 0.0001*	***D. littoralis***	**−14.0**	*P* = 0.0003
*D. lacteicornis*	35.8	*P = 0.0001*	*D. lummei*	8.4	*P = 0.0311*
*D. malerkotliana*	11.8	*P = 0.0025*	*D. mayaguana*	16.6	*P = 0.0001*
*D. mayri*	12.0	*P = 0.0001*	*D. mercatorum*	25.5	*P = 0.0001*
*D. miranda*	24.6	*P = 0.0001*	*D. mulleri*	18.8	*P = 0.0001*
*D. nebulosa*	16.8	*P = 0.0001*	*D. navojoa*	27.6	*P = 0.0001*
*D. parabipectinata*	11.8	*P = 0.0017*	*D. pachea*	56.4	*P = 0.0001*
*D. paralutea*	9.2	*P = 0.0172*	*D. pallidipennis*	11.8	*P = 0.0024*
***D. paulistorum***	**−10.8**	*P = 0.0054*	*D. palustris*	14.0	*P = 0.0003*
*D. pectinifera*	34.1	*P = 0.0001*	*D. phalerata*	14.0	*P = 0.0002*
*D. persimilis*	58.7	*P = 0.0001*	*D. polymorpha*	8.0	*P = 0.0001*
*D. phaeopleura*	31.3	*P = 0.0003*	*D. repleta*	44.0	*P = 0.0001*
*D. prostipennis*	11.4	*P = 0.0057*	***D. robusta***	**−16.0**	*P = 0.0001*
*D. pseudoananassae*	16.2	*P = 0.0002*	*D. rubida*	5.3	*P = 0.0069*
*D. pseudoobscura*	39.0	*P = 0.0001*	***D. virilis***	**−25.6**	*P = 0.0032*
*D. punjabiensis*	10.2	*P = 0.0086*	*H. pictiventris*	41.1	*P = 0.0001*
*D. rufa*	21.6	*P = 0.0001*	*S. anomala*	14.2	*P = 0.0002*
*D. sechellia*	34.8	*P = 0.0001*	*Z. indianus*	9.8	*P = 0.0115*
*D. serrata*	21.8	*P = 0.0001*	*Z. lachaisei*	13.2	*P = 0.0006*
*D. simulans*	10.2	*P = 0.0061*	*Outgroup*		
*D. suzukii*	18.9	*P = 0.0001*	*C. amoena*	20.8	*P = 0.0112*
*D. takahashii*	13.6	*P* = 0.0004	*C. procnemis*	71.6	*P = 0.0001*
*D. tani*	21.5	*P = 0.0001*	***S. pattersoni***	−10.0	*P = 0.0078*
*D. tolteca*	20.5	*P = 0.0176*			
*D. triauraria*	8.6	*P = 0.0206*			
*D. tsacasi*	26.6	*P = 0.0001*			
*D. varians*	21.5	*P = 0.0136*			
*D. vulcana*	9.8	*P = 0.0045*			
*H. duncani*	14.6	*P = 0.0002*			

### Chromosome count *vs.* genome size analyses

Female whole genome size, male whole genome size, and intersexual difference were not found to be significantly related to chromosome count (Figure S2, Regression: *P* = 0.78, *P* = 0.92, *P* = 0.271, respectively). When analyzed with Phylogenetic ANOVA, there were still no significant relationships between chromosome number and whole genome size or intersexual difference (female genome size *P* = 0.877, male genome size *P* = 0.798, intersexual difference *P* = 0.708). When repetitive content identified by soft-masking in genome sequences for 29 species is compared to their respective genome size, there is a significant relationship (*P* = 0.0398). However, there was relationship between chromosome number and repetitive content (Repeat% ∼ Chromosome Number, *P* = 0.123). When analyzed together (Repeat% ∼ GS + Chromosome Number + GS*Chromosome Number), no significant interaction between genome size and chromosome number was found (GS*Chromosome Number, *P* = 0.8462).

### Evolutionary model analyses

#### Genome size evolution*:*

The Ornstein-Uhlenbeck model, which simultaneously considers selection and drift, performed better than the White-noise and Brownian motion models when comparing models of continuous trait evolution (OU, BM, and White) in all *Drosophila* species with female whole genome size, and with male whole genome size (Table S3). OU values of α and σ^2^ for females and males across the genus are remarkably similar, suggesting there no notable differences between whole genome size evolution between females and males (Table S4).

Pagel’s parameters of evolution found evidence for strong phylogenetic signal (λ = 0.8337 in Females, λ = 0.8637 in Males, Table 3) and mostly gradual change along branches (κ = 0.855 in Females, κ = 0.8382 in Males, Table 3). Pagel’s δ was found to be 2.999 for all trait tests (Table 3), suggesting that the rate of change increased throughout time.

**Table 3:** Estimates of Pagel’s Parameters for Genome Size, and Sex Difference. λ values range from 0-1, in which 1 is complete phylogenetic signal. κ values range from 0 to 1, in which values closer to 0 indicate more punctuated change and values approaching indicate gradual change along branches. δ values can range from 0 – 3, where 1 is gradual change along the tree and values higher than 1 indicate change is occurring later in the phylogeny, near the tips

Female Genome Size			
	All Species	*Drosophila*	*Sophophora*
λ	0.834	0.514	1
κ	0.855	0.576	1
δ	2.999	2.999	2.999
Male Genome Size			
	All Species	*Drosophila*	*Sophophora*
λ	0.864	0.586	0.997
κ	0.838	0.612	1
δ	2.999	2.999	2.999
Sex Difference			
	All Species	*Drosophila*	*Sophophora*
λ	0.691	0.502	0.445
κ	0.328	0.579	0
δ	2.999	2.999	2.999

**Figure 4:**
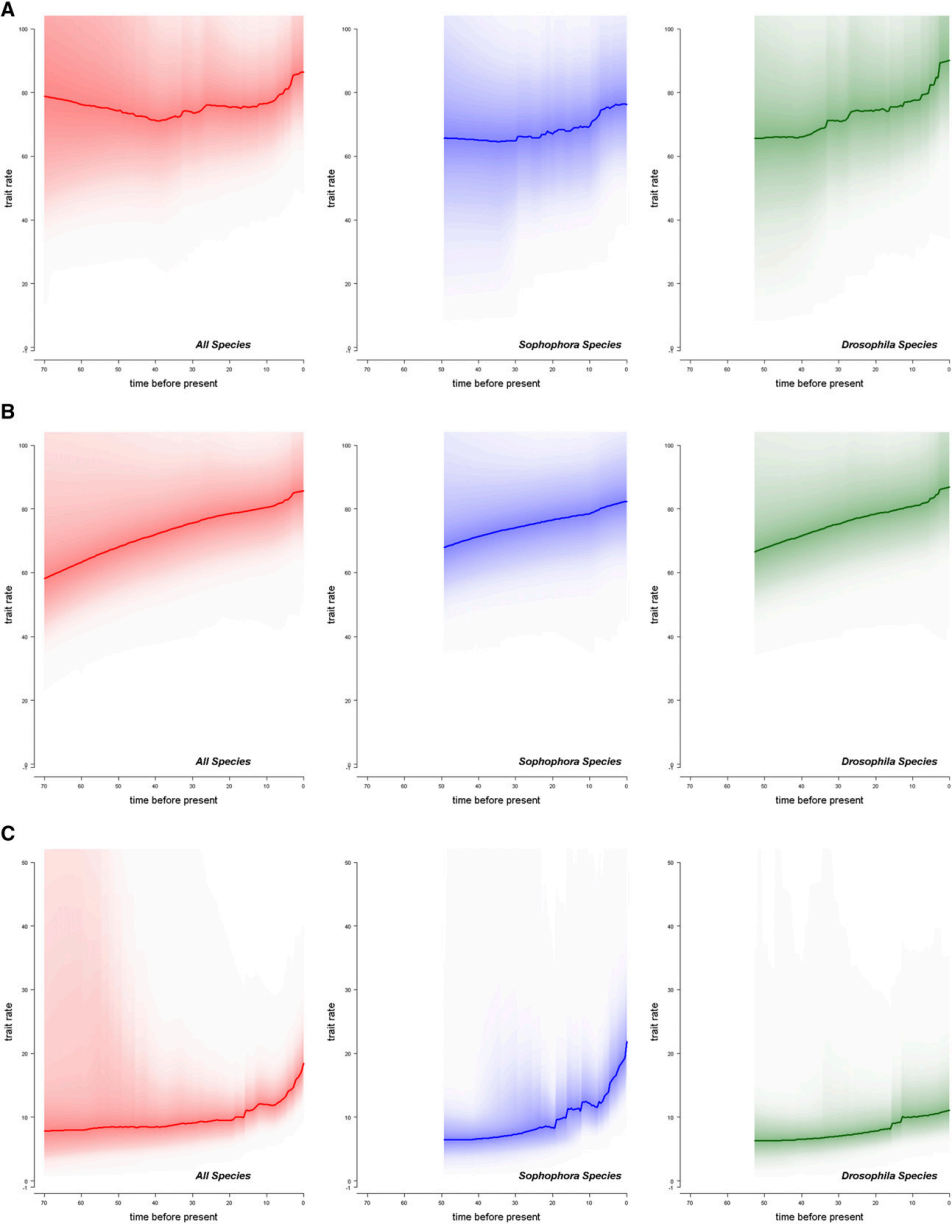
Rate of change in genome size and sex difference change through time. (A) The rate of female genome size change throughout time (B) The rate of male genome size change throughout time (C) The rate of sex difference change throughout time. The left panels (red) are for the entire *Drosophila* genus. The middle panels (blue) represent the *Sophophora* subgenus. The right panels (green), represent the *Drosophila* subgenus.

BAMM analyses on female and male whole genome size evolution find that there is a higher rate of change in recent evolutionary time (Figure 4A &4B), supporting the δ values found in the above analyses. The mostly likely number of rate shifts for female and male whole genome size in the *Drosophila* genus is 0 (Table 4). When inspecting the location of potential rate shifts, however, there appears to be more evidence for rate shifts in genome size evolution to occur primarily in the *Drosophila* subgenus (Figures S3-S6), suggesting different patterns of change may be occurring between the subgenera. This is supported visually by the higher increase in rate in the *Drosophila* clade in Figure 4A & 4B.

**Table 4:** Top ten estimated number of rate shifts estimated by BAMM. Credible shift sets were estimated for female and male genome size and difference in genome size between the sexes. Each shift set has a specific pattern of rate shifts, which may be the same or different number of shifts. Figures depicting the location of these probably shifts are in the supplementary material (Figures S3-S8)

	Female GS		Male GS		Sex Difference	
Rank	Probability	Number of Shifts	Probability	Number of Shifts	Probability	Number of Shifts
1	22.21%	0	54.34%	0	0.620%	3
2	8.01%	1	9.27%	1	0.506%	5
3	4.33%	2	3.55%	1	0.420%	6
4	3.20%	1	2.15%	2	0.341%	4
5	2.35%	3	1.49%	1	0.330%	4
6	1.11%	4	1.49%	1	0.276%	5
7	1.09%	3	1.22%	1	0.273%	4
8	1.07%	3	1.19%	1	0.257%	5
9	0.97%	3	1.01%	1	0.244%	6
10	0.88%	3	0.98%	2	0.239%	6

If the genus is separated into its two subgenera, the models of trait evolution (OU, BM, White Noise) for female and male whole genome size are consistent with those for the entire genus, with highest performance in the OU model. (Table S3). While α values for OU are similar between the *Sophophora* and *Drosophila* females and males, there is an increase in the σ^2^ estimated for genome size evolution in *Drosophila* compared to *Sophophora*, suggesting higher rates of change in the *Drosophila* subgenus (Table S4). The difference in patterns of change for genome size between the subgenera is further supported by the resulting Pagel’s parameters. There is evidence for very strong phylogenetic signal (λ = 1 for females, λ = 0.997 for males) and gradualistic change across branches (κ = 1 for females and males) in *Sophophora* (Table 3). These patterns contrast with a reduction in strength of phylogenetic genetics signal (λ = 0.514 in females, λ = 0.586 in males) and are evidence for more punctuated change on branches (κ = 0.576 in females, κ = 0.612 in males) in the *Drosophila* subgenus (Table 3). The differences in change between the subgenera in female and male whole genome size change can be visualized on a colorized trait phylogeny (Figures S9 & S10).

#### Intersexual difference*:*

Phylogenetic model testing of the difference in genome size between the sexes in each species (intersexual difference) found that OU was also the best fitting model (Table S3) with α = 0.0868 and σ^2^ = 40.97 (Table S4). Pagel’s parameters found evidence for incomplete phylogenetic signal in intersexual difference (λ = 0.691) and somewhat punctuated change on branches (κ = 0.328) (Table 3). Pagel’s δ values suggested an increase in the rate of change throughout time (δ = 2.999, Table 3).

BAMM analyses further support the increase in rate for intersexual difference in genome size change in recent history (Figure 4C). There is low support for any number of shifts throughout the phylogeny, suggesting heterogeneity in the rate of change in sex difference across the phylogeny. While there is not necessarily one pattern of rate shifts that is more frequent than others, the highest probability is for three shifts (0.62%) (Table 4). If we look over all of the posterior, the number of rate shifts which has the highest probability is 9 (11.5%). When inspecting these potential rate shifts, a large proportion of these rate shifts in sex difference occur in the *Sophophora* subgenus (Figures S7 & S8), suggesting that the patterns of change in sex difference differs between the subgenera.

When intersexual difference is separated by subgenus, the OU model continues to be the best fitting model tested, with similar support for the White-noise model in *Sophophora* but not in *Drosophila* (Table S3). While there is similar support for the OU model in both subgenera, there are increased α and σ^2^ values in *Sophophora* compared to the *Drosophila* subgenus (*Sophophora* α = 0.2353, σ^2^ = 83.494; *Drosophila* α = 0.0378, σ^2^ =14.665) (Table S4). The increase in σ^2^ in *Sophophora* could result from the larger intersexual differences found in that subgenus (Table S4, Figure 2).

Pagel’s λ and δ were similar between the subgenera (*Sophophora* λ = 0.445, δ = 2.999; *Drosophila* λ = 0.5019, δ = 2.999, Table 3); however, there was a large reduction in the κ value found for *Sophophora* compared to *Drosophila*, suggesting a more punctuated change in intersexual difference on branches in *Sophophora* than *Drosophila* (*Sophophora* κ = 0, *Drosophila* κ = 0.5787, Table 3). The differences in sex difference change between the subgenera can be visualized on a colorized trait phylogeny (Figure 1).

We used absolute intersexual differences for these analyses; however, we wondered if accounting for the proportion of the genome size represented by the intersexual difference would produce different results. A 10 Mbp difference could have a much larger impact in a species with a 200 Mbp genome than one with a 300 Mbp genome. In order to normalize, absolute intersexual difference was divided by the average of the diploid male and diploid female genome size. All tests were performed on these normalized values. While using normalized values produced quantitatively different results (percentages of the genome are much smaller than absolute differences), they did not produce qualitatively different results.

## Discussion

We report new and updated female and male genome size estimates for 92 species of *Drosophila* and related genera (53 new species genome size records, 39 updated species genome sizes), with a focus on the *Drosophila* subgenus, including *Zaprionus*. The added values dramatically increase the representation of the *Drosophila* subgenus to approximately equal estimates to *Sophophora*. The increase in estimates allow us to investigate patterns of change in a total of 152 species. Genome size varies extensively across the species studied, from 137.5 Mbp in male *D. bromeliae* to 395.2 in female *C. amoena* –a nearly a threefold range (Table S2). No significant difference was found between genome sizes of *Sophophora* and *Drosophila* when comparing female or male whole genome sizes. It is important to recognize that the species in Table S2 are represented by a single strain, and the conclusions are strictly valid only for those strains. We do not know if any of the conclusions would differ if we had scored different strains, but have reason to believe the differences would be few. We scored 200 inbred strains of *D. melanogaster* (Huang *et al.* 2014) and found that a single strain is very unlikely to deviate far from the species average. The average across 200 strains of *D. melanogaster* strains was 175.5 Mbp, which is within 0.5 Mbp of the value generally reported for that species. All but four of the 200 strains fell between 170 and 180 Mbp. The four strains outside that range had genomes larger than 180 Mbp, which accounted for the slightly large average for the species.

We find no relationship between genome size and chromosome number in the *Drosophila* species we have investigated, with or without accounting for phylogenetic relationships. The lack of relationship is supported by a recent study which found that genome size correlates to chromosome size, but not chromosome number, in snapping shrimp (Jeffery *et al.* 2016). The above results differ from previous studies, which found that chromosome number in angiosperms (890 species from 62 genera) (Pandit *et al.* 2014) is positively correlated to genome size . This pattern was also suggested in early studies in vertebrates where diploid teleost fishes had a significant positive correlation between genome size and chromosome count (Hinegardner and Rosen 1972). However, while there is much evidence for this trend, this pattern has not been clearly supported across other taxa. In plants the pattern is often contradictory: genome size has been found to correlate to chromosome count in *Carex* (Escudero *et al.* 2015), yet this correlation was not maintained in *Ginlisea*, a carnivorous plant genus (Fleischmann *et al.* 2014) or cycads (Gorelick *et al.* 2014). In that regard, these *Drosophila* results fit with other arthropods and some plants, but do not support the results found in fish and some plants.

Chromosome number can change through a variety of processes. The most extreme of these is whole genome duplication, in which the genome size may be dramatically increased. The relationship between genome size and chromosome number in angiosperms and teleost fish is likely due to these polyploidy events (Hinegardner and Rosen 1972; Pandit *et al.* 2014). The events following these polyploidization events typically result in duplicate gene losses and chromosomal rearrangements which may make these events difficult to identify, and may remove any signal of a relationship between genome and chromosome number (Reviewed in Wright 2017). Chromosome number can also change by fusions or fissions of existing chromosomes, changing chromosome number but not the actual number of chromosome arms (fundamental number) or genic content of the genome (Blackmon *et al.* 2019). Similar fissions and Robertsonian fusions are likely what is occurring in these *Drosophila* species. Fusions or fissions may not lead to doubling of the genome size, but they can change the number of structural regions of the genome (number of centromeres or telomeres). Our results suggest that changes in the number of structural elements associated with fusions and fissions has insufficient impact on the genome size to detect with the methods that we have applied. At this point, there still remains no clear support for a consistent pattern between genome size and chromosome number, except in regard to polyploidy events (Elliott and Gregory 2015; Slijepcevic 2018).

The patterns of absolute genome size across the *Drosophila* genus do not appear to differ between females and males when accounting for phylogenetic relationships. When investigating the genus as a whole there is strong phylogenetic signal and gradualistic change (Female λ = 0.834, κ = 0.835). In support of this, BAMM analyses finds that the most likely number of rate shifts in genome size change is zero. These results support previous work on genome size evolution in the *Sophophora* subgenus of *Drosophila* (Hjelmen and Johnston 2017; Hjelmen *et al.* 2019). Interestingly, further investigation into potential rates shifts in genome size highlights specific clades and species which seem to break this pattern of gradualistic change (Figures S3-S6). For example, there are predicted rate shifts for *D. suzukii* and *D. orena* species in the *Sophophora* subgenus. These species both seem to have had genome expansions compared to the closely related species: *D. suzukii* having a genome size of 342.8 Mbp compared to species in the same clade ranging from 210-245 Mbp, and *D. orena* having a genome size of 280.7 Mbp compared to *D. erecta* with a genome size of 184 Mbp (Table S2).

While the above two species exhibit dramatic expansions of whole genome size, many more potential rate shifts are located in the *Drosophila* subgenus (Figures S4 & S6). There is for example support for a high rate of genome size change within the *Drosophila* clade containing *D. kohkoa* and *D. albomicans* (Figures S4 & S6). This clade seems to have largely increased in genome size from the sister clade of *D. rubida* and *D. hypocausta* (218.7 and 190.6 Mbp, respectively, Table S2). The clade of interest ranges from 215.9 Mbp to 271.9 Mbp, with the exception of *D. neohypocausta*, which has been reduced to 165.7 Mbp (Figures S4 & S6, Table S2). The dramatic shifts upwards and downward suggest large amounts of change in this clade. Another exceptional clade worth noting is the one containing *D. pallidipennis* and *D. tripunctata*. The species in that clade range from 179.2 to 330.9 Mbp, with no clear phylogenetic separation of species with large and small genomes (Table S2). Further analysis of these two clades with brownie.lite found significantly higher rates of evolution in these clades compared to the rest of the phylogeny (*P* = 0.02). The large shifts in genome size in these two clades suggest that whole genome size may be evolving differently in the *Drosophila* subgenus.

When the *Sophophora* and *Drosophila* subgenera are compared with phylogenetic comparative methods, there are differences found in the patterns of whole genome size change. While there are no differences in the average genome sizes between the subgenera (*t*-test, *P* > 0.05) and similar alpha values from tests of OU (Table S4), there is a larger σ^2^ value in the *Drosophila* subgenus than the *Sophophora* subgenus, suggesting a higher rate of genome size change among the *Drosophila* subgenera species. There is also a notable reduction in phylogenetic signal and reduction in κ, suggesting less similarity among related species and more punctuated change in the *Drosophila* subgenus when compared to *Sophophora* (λ = 0.514 *vs.* 1, κ = 0.576 *vs.* 1, respectively, Table 3). These results suggest that the 40-65 million years since the divergence of these two subgenera have had an impact on the patterns of whole genome size change, yet the impact is not related to their karyotypic differences. Therefore, it is important to note that patterns of genome size change may differ between closely related organisms with different evolutionary pasts. There have been other reported differences in genome size patterns within an order. There was no phylogenetic signal in seed beetles and strong evidence for phylogenetic signal and change by neutral processes in fireflies, both species within the order Coleoptera (Arnqvist *et al.* 2015; Lower *et al.* 2017); this may be the first reports of genome size patterns differing between subgenera.

Over half of the species in this study were found to have significant differences between estimates of female and male genome size (83 species, Table 2). Forty-five of these species were found in the *Sophophora* subgenus, 35 within *Drosophila*, and three in outgroups Table 2). Because *Drosophila* males have achiasmatic meiosis, the entire Y chromosome is non-recombining and experiences a range of population genetic forces (*e.g.*, Muller’s ratchet, background selection, ruby in the rubbish, etc.) that are expected to lead to the reduction in functional genic content (Peck 1994). Often due to deletion biases, this eventually leads to a reduction in the physical size of the non-recombining chromosomes (Sundström *et al.* 2003). However, there are exceptions to this where the Y chromosome expands in size due to the expansion of repetitive elements such as transposons (Zhou *et al.* 2012).

In the majority of cases in this manuscript, the female is found to have a larger genome than the male. Eleven species were found to have males with statistically significant larger genomes than the females. Nine of these species were found in the *Drosophila* subgenus, most notably *D. albomicans*, *D. robusta*, and *D. lacertosa*, all of which have reports of neo-sex chromosome systems (Flores *et al.* 2008). *D. kepulauana* is closely related to *D. albomicans*, suggesting similarity in sex chromosome system. Others of these species, such as *D. littoralis*, *D. virilis*, and *D. ezoana* are of the *virilis-robusta* expansion in which there are reported instances of neo-sex chromosomes (Flores *et al.* 2008).

While there were no remarkable differences in patterns of whole genome size evolution between the sexes in either subgenus, significant differences were found between the subgenera when investigating the difference in genome size between the sexes - a proxy for the differentiation of the sex chromosomes (*Sophophora* = 13.9 Mbp, *Drosophila* = 5.1 Mbp, *t*-test *P* = 0.0001 Figure 2). The positive values for the difference indicate that female genomes, on average, are larger than the male, which is not surprising given previous work on intersexual genome size differences in this genus (Hjelmen *et al.* 2019). However, these data suggest that the difference in sexes is larger in *Sophophora* than in *Drosophila*. This difference could be due to differing numbers of chromosomes, with the sex chromosomes making up different proportions of the genome; yet, there was no significant relationship between intersexual difference and chromosome number (*P* > 0.05). One potential explanation for the lack of a significant relationship between chromosome number and intersexual difference is that some species with fewer chromosome have fusions among autosomes that are unlikely to impact sex difference, while others have fusions between autosomes and sex chromosomes which are likely to impact sex difference. Further, when investigating the sex difference among species known to be XO, XY, and neo-sex, there were no differences between XY and neo-sex species (GLM, *P* = 0.140), yet XO species not surprisingly had significantly larger sex differences than the other sex systems (GLM, *P* < 0.0001). It is important to note that neo-sex chromosomes can be young and undifferentiated with likely little intersexual difference, or old and differentiated with a larger intersexual difference. Therefore, not only may there not be significant differences between XY systems and neo-sex systems, but it can also be difficult to identify neo-sex systems without chromosome synteny information. For a handful of species, the Muller elements that have been incorporated into the sex chromosomes is known (Blackmon and Demuth 2015). Documenting the Muller element content of the sex chromosomes in more species with sex differences in genome size may provide greater insights into trends in genome size divergence as sex chromosomes evolve.

Intersexual difference across the phylogeny was found to have reduced phylogenetic signal and more punctuated change (λ = 0.691, κ = 0.328, Table 3). The *Sophophora* subgenus was found to exhibit increased rates of change in recent time when compared to *Drosophila* (Figure 4A &4B). OU α and σ^2^ values were higher in *Sophophora* than *Drosophila*, suggesting larger variance in sex differences and a larger magnitude of deviation in sex difference size than in *Drosophila* (Table S4). This difference in the subgenera is further supported by a reduction in phylogenetic signal and punctuated change in *Sophophora* according to Pagel’s parameters of evolution when compared to *Drosophila* (Table 3). According to BAMM analyses, a large proportion of the mostly likely locations for rate shifts in sex differences were found in clades within the *Sophophora* subgenus (Figures S7 & S8). Many of these likely rate shift locations are located in clades with known neo-sex chromosomes. For example, there is high support for rate shifts in the clades containing *D. pseudoobscura*, *D. miranda*, and *D. albomicans*, all of which are well studied due to their known sex chromosome turnovers (Mahesh *et al.* 2000; Bachtrog 2004; Carvalho and Clark 2005; Bachtrog *et al.* 2008; Lin *et al.* 2008; Cheng *et al.* 2011). Support for rate shifts has also exists for the *virilis-repleta* radiation, another group known for sex chromosome turnover (Flores *et al.* 2008). A final clade to note is the *nannoptera* group of *Drosophila*. These cactophilic *Drosophila* are noted for their unique life-history characteristics and increased heterochromatin content (Heed and Kircher 1965; Ward and Heed 1970). One species of note in this clade is *D. nannoptera*, which exhibits evidence for Y-specific genes shifting to autosomes (Dupim *et al.* 2018) and a slightly, but not significantly, larger male genome size than female genome size (Table S2). The sex difference results support somewhat unpredictable turnovers in sex chromosomes, and substantial differences in genome size between the sexes. Punctuated change in sex chromosomes (low κ value) are likely to coincide with speciation events, in which hybridization may be less likely between newly diverged species. These patterns of speciation with neo-sex systems has been supported by work in sticklebacks (Kitano *et al.* 2009).

In conclusion, patterns of whole genome size evolution differ, not only between species within the same order (Arnqvist *et al.* 2015; Lower *et al.* 2017), but also within more closely related groups such as subgenera. While the 40-65 million years of evolution that has passed since the divergence of *Drosophila* and *Sophophora* has resulted in no difference in whole genome size on average, the patterns by which genome size has changed in each subgenera differs, with much more predicable phylogenetic patterns within the *Sophophora*. While there are karyotypic changes throughout this genus which will impose changes in chromatin structure and genome architecture, there is no effect of this karyotypic change on genome size. This lack of effect is not unexpected, but can be disentangled further with future investigations of heterochromatin’s effect on genome size evolution. These differing results suggest that genome size evolution in the *Drosophila* subgenus may not be explained by the accordion hypothesis (Kapusta *et al.* 2017), but rather another hypothesis. The above results warrant further investigation into mobile elements, repetitive sequences, and other structural changes in the genome within the clades of *Drosophila* which exhibit high rates of change, such as the *nannoptera* group, *D. pallidipennis*, *D. kohkoa*, etc. which may be associated with these large shifts.

Many questions remain. Are there life history and/or environmental factors that may be acting on phenotypic correlates of genome size? Are there population genetic effects influencing these dramatics shifts in the *Drosophila* subgenus that are experienced less in the *Sophophora* subgenus (Powell 1997; Lynch and Conery 2003; Gregory and Johnston 2008)? Remarkably, while there are no differences in genome size or patterns of change between whole genome size of sexes in *Sophophora* and *Drosophila*, there are significant differences in patterns by which sexes differentiate in genome size, suggesting differences in sex chromosome evolution. While much of the rate heterogeneity of sex differences may be explained by the incidence of neo-sex chromosomes, the clades of *Drosophila* with changes in rates must be further investigated to give a more complete story of the incidence of sex chromosome turnover and Y chromosome degradation.
